# SIP-Based Single Neuron Stochastic Predictive Control for Non-Gaussian Networked Control Systems with Uncertain Metrology Delays

**DOI:** 10.3390/e20070494

**Published:** 2018-06-26

**Authors:** Xinying Xu, Yalan Zhao, Mifeng Ren, Lan Cheng, Mingyue Gong

**Affiliations:** College of Information Engineering, Taiyuan University of Technology, Taiyuan 030024, China

**Keywords:** networked control systems, data-driven predictive strategy, non-Gaussian random delays, non-Gaussian disturbances, survival information potential

## Abstract

In this paper, a novel data-driven single neuron predictive control strategy is proposed for non-Gaussian networked control systems with metrology delays in the information theory framework. Firstly, survival information potential (SIP), instead of minimum entropy, is used to formulate the performance index to characterize the randomness of the considered systems, which is calculated by oversampling method. Then the minimum values can be computed by optimizing the SIP-based performance index. Finally, the proposed strategy, minimum entropy method and mean square error (MSE) are applied to a networked motor control system, and results demonstrated the effectiveness of the proposed strategy.

## 1. Introduction

In the last decade, network technology has dramatically been improved. More and more control systems are combined with network technologies [1,2,3]. Networked control systems (NCSs) are loop-locked control systems in which a closed loop is constituted by communication channels [4,5]. In fact, control system with communications is not a new concept in automation. From tele-operation to distributed control, control theory over a communication network has already been developed for more than 40 years. However, there are many factors that distinguish the current NCSs and previous control systems with communications. Two of them are the most significant: (1) in the previous control with communications, the network was specialized and dedicated for the stability of process operation and timeliness of information exchange, while in the current NCSs the network is general-purpose and public for various concurrent applications, and thus stable operation and real-time communication are no longer ensured; (2) their functionality has been diversified tremendously, and a variety of control and management or administrative functions have been developed, which is different from previous single control [6]. As a new area of control systems, NCSs have advantages of resource sharing, achieved remote monitored and adjusted, low cost, simple installation and high reliability [7,8], which has been applied to many fields such as tele-surgery, tele-manufacturing, museum guidance, space exploration, traffic control, health care and disaster rescue [9], but demands on complexity, diversity, and real-time performance for networked operations have brought new technological challenges to NCSs. Time delays exist inevitably in the communication net [10]. When sampling periods are longer than these delays, the influence of the delays is need not to be considered in general control systems. However, the network control system has high requirements on real-time, so delays induced via network communication are becoming more and more important to be analyzed and designed in the NCSs [11,12]. What’s more, the occurrence of disturbances can also degrade their control performance [13]. Therefore, the control of stochastic NCSs drew in large studies. 

The random network delay only in the forward channel in NCSs was considered in [14]. However, delays usually exist both in the forward and feedback channels, which make the control design and stability analysis much more challenging. In order to solve this problem, [15] proposed a control scheme for NCSs with random network delays in both channels, in which the model of random network-induced delays is utilized by Markov chains. In addition, a model-based predictive control algorithm was developed by Liu et al. [16,17] with the purpose of better control. However, these methods were based on the model. Moreover, the shortcomings of the accurately model building were overcome by data-driven predictive control method [18].

The above presented control methods only considered the random delays in NCSs, it is known that external disturbances are also inevitable in NCS, which may lead to the deterioration of control performance. One- and two-parameter control schemes were researched to track the performance of continuous systems with white Gaussian disturbances in [19,20]. The result was further generalized to other noisy channels of NCSs in [21], where achievable minimal tracking error is used to measure optimal tracking performance. However, these methods are mostly based on the assumption that the system variables follow a Gaussian distribution. Under such an assumption, all possible statistical information of a variable can be extracted from its mean and variance. In fact, most network systems are difficult to meet these pre-conditions because of the mixture of nonlinear factors or different disturbances, which results in non-Gaussian randomness even if the disturbances are of Gaussian types. Stochastic distribution control theory, developed by Wang [22], had been applied to the control of stochastic systems with non-Gaussian disturbances [23,24]. Based on this theory, Zhang et al. applied minimized zero mean entropy method to NCSs, and the corresponding controller was designed [25]. In [26], fault detection of networked control systems via minimum entropy observer was presented and in [27], a minimum entropy Luenberger observer was designed for linear time-invariant (LTI) system and Van der Pol Oscillator. However, system models in the studies mentioned above were all linear. As we all know, in practical engineering, nonlinear systems generally exist, and most of them meet the Lipschitz condition. Ren et al. [28] proposed an entropy-based control algorithm for nonlinear NCSs with non-Gaussian random disturbances and delays. Actually, some drawbacks exist in entropy: (1) it is shift-invariant (i.e., its value remains unchanged even if the location of distribution varies); (2) the value may be negative; (3) When PDF is non-existent, the definition will be ill-suited. In order to overcome these drawbacks, a single neuron control strategy for non-Gaussian stochastic systems based on the survival information potential (SIP) criterion was proposed by Chen et al. [29], in which control input was conservatively considered as a deterministic variable [28,30]. In fact, the randomness of control input exists in practical conditions, so paper [31] proposed a single neuron stochastic predictive PID control algorithm using SIP criterion, in which the performance index not only contains the SIP of tracking error, but also includes the SIP of control input. And it is for nonlinear systems with exact model. But it is usually difficult to obtain accurate model of NCSs. What’s more, the random delays are inevitable in NCSs. Therefore, a data-driven single neuron predictive control structure is constructed by SIP for NCSs with non-Gaussian disturbances and random delays in this paper.

This paper is organized as follows: in Section 2, the control problem of the NCSs with non-Gaussian disturbances a time delays is formulated. Specifically, the performance index of SIP is formulated. Based on the proposed performance index, the single neuron stochastic predictive control method is mentioned in Section 3. The efficiency of the proposed control strategy is illustrated through applying method to networked motor control system in Section 4. The last section is a summary of this paper. 

## 2. Problem Formulation

NCSs are dynamic systems and each control loop is closed via a network communication channel, which induces possible random delays. It is known that random disturbances are inevitable in NCSs. Both factors may affect NCSs’ control performance and stability. What’s more, there are multiple unknown parameters and uncertainties in the NCSs, the system has strong nonlinear characteristic, and its model is difficult to be established. Using the equivalent model and ignoring a number of uncertainties are common solutions, which would greatly reduce the accuracy of model and seriously affect the control effect. Data-driven method just need the process data before the algorithm started, rather than the modeling of the process. The data-driven control model for the NCSs can be expressed as:

(1) $$y_{k}=f(u_{k},\omega_{k},d_{k},\tau_{k})$$

where $y_{k}$ and $u_{k}$ are system output and control input. $\omega_{k}$ is a kind of random non-Gaussian disturbance. The independent signals, $d_{k}$ and $\tau_{k}$, are random and can be described by the beta distribution respectively, their values are between 2 and 8.

Based on the process described of NCSs above, the tracking error is:

(2) $$e_{k}=f(r_{k},u_{k},\omega_{k},d_{k},\tau_{k})$$

where $r_{k}$ is the set point. $e_{k}$ can be viewed as a function of $d_{k}$, $\tau_{k}$ and $\omega_{k}$.

**Remark** **1.** f(⋅) *and* g(⋅) *are unknown functions. The model is needless in the proposed method, Equations (1) and (2) are utilized to depict the relations between and among error, disturbance and delays.*

**Figure 1:**
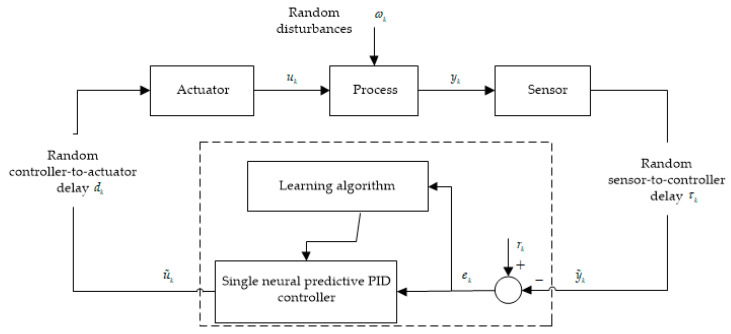
The schematic of NCSs with single neuron predictive controller.

In NCSs, the plant, controller, sensor, actuator and reference command are connected through a network [32]. The scheme of NCSs is shown in Figure 1. Bounded random delays $\tau_{k}$ and $d_{k}$ exist in both channels from sensor to controller and controller to actuator respectively. The control input ${\tilde{u}}_{k}$ from single neuron predictive controller could be transferred to actuator after $d_{k}$ time. And then, the executive action $u_{k}$, $u_{k}={\tilde{u}}_{k+d_{k}}$, is utilized to control the process and then output $y_{k}$ can be obtained. The purpose of this paper is to design a single neuron predictive controller to make the tracking error $e_{k}$, $e_{k}=r_{k}-{\tilde{y}}_{k}$, approach to zero as closely as possible, where ${\tilde{y}}_{k}$ is the measured output, ${\tilde{y}}_{k}=y_{k+\tau_{k}}$.

The single neuron controller is in use here. It is a nonlinear processing unit with multiple inputs and single output, which can be shown as Equation (3):

(3) $$u_{k}=u_{k+1}+K\sum_{l=1}^{3}\omega_{lk}x_{lk}/\left\|\Sigma_{k}\right\|$$

where $\sum_{k}=\sum_{l=1}^{3}\omega_{lk}$, K>0 is the proportional coefficient of the neuron. $\omega_{lk}(l=1,2,3)$ is the weight corresponding to each input $x_{lk}(l=1,2,3)$:

(4) $$\{\begin{array}{c} x_{1k}=e_{k}\\ x_{2k}=e_{k}-e_{k-1}\\ x_{3k}=e_{k}-2e_{k-1}+e_{k-2} \end{array}$$

In Figure 1, delays $d_{k}$, $\tau_{k}$ and disturbance $\omega_{k}$ follow non-Gaussian distributions. According to the statement above, Gaussian randomness of system could be sufficiently characterized by mean and variance (covariance), which are unavailable for systems with non-Gaussian randomness. Therefore, a criterion should be used to deal with non-Gaussian randomness. 

## 3. SIP-Based Performance Index

### 3.1. Overview of Entropy Criterion

In the previous works, MSE can control well under the assumption that system variables are of Gaussian type, but it is not suitable for non-Gaussian randomness. Entropy is usually chosen for the performance index to reject non-Gaussian randomness of systems. There are many kinds of the entropy [33], but Shannon entropy certainly plays an important role. For a continuous random variable X with PDF $\gamma_{X}(x)$, the definition of Shannon entropy is described:

(5) $$H_{S}(X)=\int_{-\infty }^{+\infty }\gamma_{X}(x)\log\gamma_{X}(x)dx$$

Then, a well-known generalization of Shannon entropy is Renyi entropy defined by:

(6) $$H_{\alpha }(X)=\frac{1}{1-\alpha }\log\int_{-\infty }^{+\infty }\gamma_{X}^{\alpha }(x)dx$$

where α is the entropy order and α>0, α≠1. It can be shown that Renyi entropy will reduce to Shannon entropy when α→1.

The argument of the log in the Renyi entropy (6) is called the information potential1 (IP):

(7) $$V_{\alpha }(X)=\int_{-\infty }^{+\infty }\gamma_{X}^{\alpha }(x)dx$$

Since Renyi’s entropy $H_{\alpha }(X)$ is a monotonic decreasing function of the IP $V_{\alpha }(X)$ when α>1, and $V_{\alpha }(X)$ is simpler for computing.

From Equation (7), it can be seen that some drawbacks exist in IP: (1) it is shift-invariant (i.e., its value remains unchanged even if the location of distribution varies); (2) the value may be negative; (3) When PDF is non-existent, the definition will be ill-suited.

### 3.2. SIP Criterion

In order to overcome drawbacks of entropy, a more general measure, survival information potential (SIP) is chosen for the performance index. According to [29], SIP can be defined as follows:

**Definition** **1.** *For a random variable $X\in {\mathbb{R}}^{m}$, SIP of order* α(α>0) *is defined by*

(8) $$S_{\alpha }(X)=\int_{{\mathbb{R}}_{+}^{m}}{\bar{F}}_{\left|X\right|}^{\alpha }(x)dx$$

*where* ${\bar{F}}_{\left|X\right|}(x)=P(\left|X\right|>x)=E\left[I(\left|X\right|>x)\right]$ *is the survival function (or equivalently, the distribution function) of the random variable* |X|*, and* ${\mathbb{R}}_{+}=\left\{x\in \mathbb{R}:x\geq 0\right\}$*.* I(⋅) *is the indicator function.*

There are some advantages that can be concluded from the definition of SIP: (1) it is not shift-invariant (i.e., its value remains unchanged even if the distribution location varies); (2) it has good robustness because of more regular distribution function; (3) it is easy to compute values from the sample data to avoid computing the kernel and choosing the kernel width.

The control input is also non-Gaussian according to the single neuron controller Equations (3) and (4), so it is improper to consider conservatively the control input as a deterministic variable. What’s more, predictive control strategy is used to obtain the optimal control input here, which has achieved well performance, as above. Both tracking error and control input are considered, the following predictive performance index J is utilized to optimize the weights of single neuron:

(9) $$J=\sum_{i=1}^{P}S_{\alpha }(e_{k+i-1})+\lambda \sum_{j=1}^{M}S_{\alpha }(u_{k+j-1})$$

where $e_{k+i}$ is the i-step ahead prediction of the tracking error relating to disturbance and delays. $u_{k+i}$ is the i-step ahead prediction of the control input. P and M are the prediction horizon and the control horizon, respectively, and M≤P. It’s worth noting that the control input would not change after M steps, i.e., $u_{k+j-1}=u_{k+M-1}(j>M)$.

It is not easy to describe the distribution of the error $e_{k}$ with integrated first-principle model in actual industry. Therefore, the data-driven empirical SIP, instead of the theoretical SIP, is used here. Suppose there are error samples $\left(e_{1k},e_{2k},\cdots ,e_{Nk}\right)$ at instant k, and without the loss of generality, that $\left|e_{1k}\right|\leq \left|e_{2k}\right|\leq \cdots \leq \left|e_{Nk}\right|$, the empirical SIP of error as follows [29]:

(10) $${\hat{S}}_{\alpha }(e_{k})=\sum_{m=1}^{N}\mu_{m}\left|e_{mk}\right|$$

where $\mu_{m}={\left(\frac{N-m+1}{N}\right)}^{\alpha }-{\left(\frac{N-m}{N}\right)}^{\alpha }$.

According to Equation (10), the empirical SIP ${\hat{S}}_{\alpha }(u_{k+m-1})$ of control input can similarly also be formulated. The performance index $\hat{J}$ with empirical SIP can be expressed as:

(11) $$\hat{J}=\sum_{i=1}^{P}\sum_{m=1}^{N}\mu_{m}\left|e_{mk+i-1}\right|+\lambda \sum_{j=1}^{M}\sum_{m=1}^{N}\mu_{m}\left|u_{mk+j-1}\right|$$

where $\mu_{m}={\left(\frac{N-m+1}{N}\right)}^{\alpha }-{\left(\frac{N-m}{N}\right)}^{\alpha }$.

The calculation of empirical SIP needs samples. Taking into account the realization of actual operation, we handle samples with an oversampling method [34].

**Figure 2:**
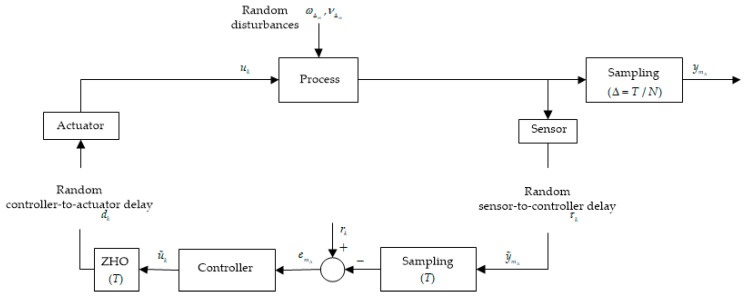
Over-sampling technique in NCSs.

Assuming the over-sampling rate is N, it is a positive integer. Figure 2 displays the structure diagram of NCSs with an over-sampling technique. Like in general closed-loop system, NCSs contain process model $G_{p}$ and controller of which the control period is T. A zero-order holder is behind controller, which is used to generate piecewise control input. It is noting that the output sampling time equals Δ=T/N, and then the signal $y_{\Delta }(m)(m=k,k+\Delta ,k+2\Delta ,\cdots ,k+(N-1)\Delta )$ can be generated, while a conventional output signal is sampled at a period of T. Specially, under the effect of zero-order holding, the input signal $u_{\Delta }(m)$ is actually generated as:

(12) $$u_{\Delta }(m)=u(k),(m=k,k+\Delta ,k+2\Delta ,\cdots ,k+(N-1)\Delta )$$

The over-sampling method is presented above for an NCS. Particularly, the value of T is one second. In simple terms, the control input is sampled at a period of T and then will be held until next sampling due to zero-order holding, but the output is sampled at a period of Δ=T/N. Therefore, N samples of output can be sampled after T time, and then, N samples of error between N samples of output and corresponding reference trajectory can be obtained. Similarly, substituting N error samples into Equations (3) and (4), N control input samples also can be obtained. After substituting N samples of error and control input into Equation (11), the performance index can be calculated through empirical SIP.

## 4. Optimal Control Algorithm

To achieve the optimal weights of the single neuron, considering the structure of the single neuron adaptive controller from Equation (3), the performance index from Equation (9) will be:

(13) $$w_{k}^{*}=\underset{w_{k}\in {\mathbb{R}}^{3M}}{\arg\min J}$$

where $w_{k}={\left[w_{1,k},w_{2,k},w_{3,k},w_{1,k+1},w_{2,k+1},w_{3,k+1},\cdots ,w_{1,k+M-1},w_{2,k+M-1},w_{3,k+M-1}\right]}^{T}\in {\mathbb{R}}^{3M}$.

There are many optimal algorithms for solving the optimal value of Equation (13), among which the gradient descent method is the earliest and most commonly used optimization method [35]. The gradient descent method is simple. When the objective function is convex, the gradient descent method is the global solution. Its idea is that make the negative gradient direction of current position as search direction, which is the fastest decline direction. Therefore, it also is known as “the steepest descent method”. Based on the gradient descent method and Equation (11), the optimal weight can be updated by:

(14) $$w_{k+1}=w_{k}-\eta \frac{\partial {\hat{J}}_{k}}{\partial w_{k}}$$

where η>0 denotes adaptation gain. 

The optimal input $u_{k}$ of NCSs can be computed according to Equation (3). The following steps describe the process of proposed control algorithm in detail:

*Step 1*: Giving initial value of weight vector $w_{0}$ and operation time $k_{\max}$. Setting parameters of step-size η and α.

*Step 2*: Calculate the performance index at present instant from Equation (11).

*Step 3*: Obtain the update weight vector $w_{k+1}$ through the gradient descent method by Equation (14).

*Step 4*: Substitute the optimal weight vector into Equation (3), and then the next control input is obtained.

*Step 5*: Process outputs could be collected according to corresponding control input, which could be used to update the next performance index. Then implement repeatedly processes from Step 2 to Step 5 for the next time step, k=k+1, until $k=k_{\max}$.

## 5. Simulation Results

**Figure 3:**
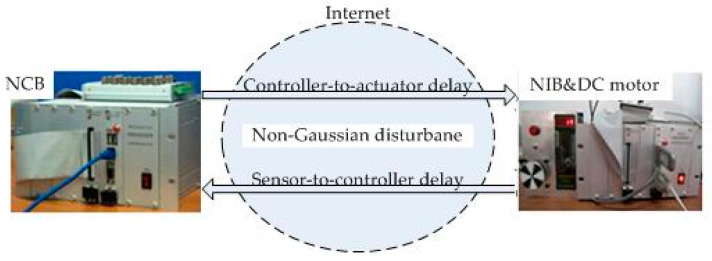
Networked DC motor control system.

A networked DC motor control test rig is utilized to demonstrate the performance of the proposed method, which consists of two NetControllers and a DC motor with its driver, as shown in Figure 3. Both the networked controller board (NCB) and networked implementation board (NIB) have the same hardware structure. NIB is on the DC motor, which is located in the Huazhong University of Science and Technology (Wuhan, China), while the NCB is located remotely at the North China Electric Power University (Beijing, China). The two parts are connected by the internet which contains non-Gaussian disturbaces and delays, and the communication protocol between them is the UDP. The test rig adopts a 57BL (3)-10-30 DC servo motor, which is made by Zhuhai Motion Control Motor Co. Ltd. The model parameters and mathematical model of the considered DC motor can be obtained from Ren et al. [28].

**Figure 4:**
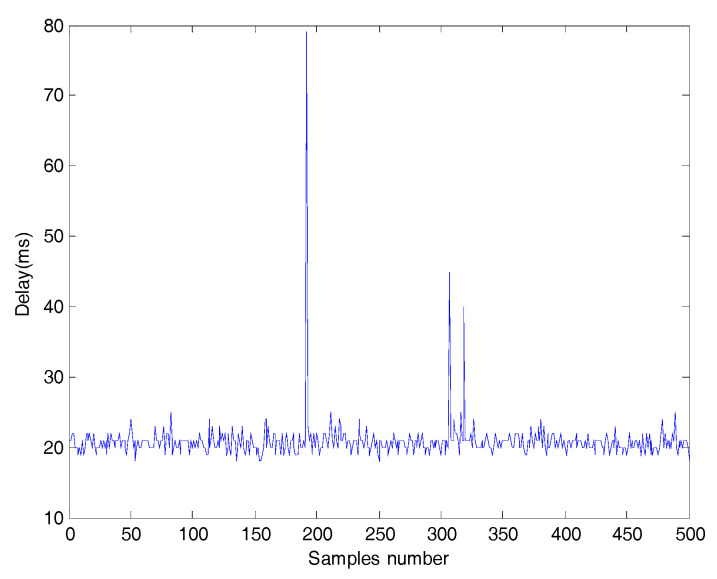
Data graph of time delays.

**Figure 5:**
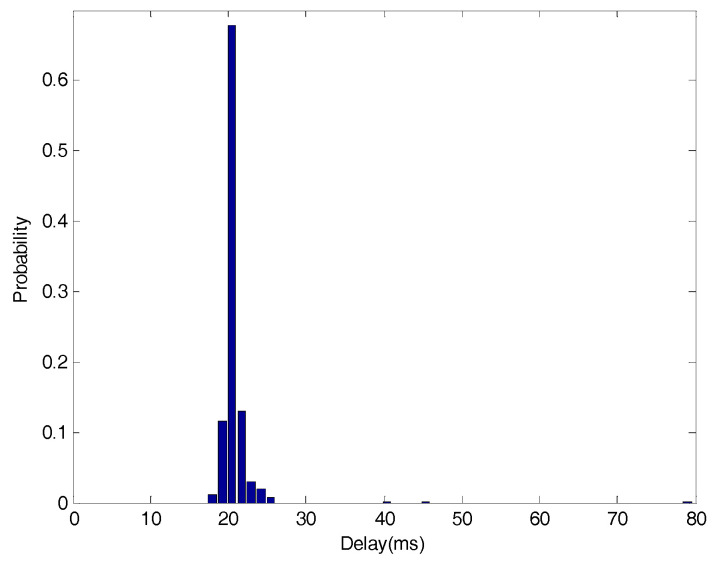
The distribution of time delays.

The goal of the control design is not only to reach the target value of rotational speed of the armature r=3000 rad/min but also to reduce the variation of the corresponding variable and random delays. Considering the convenience of analysis, the round trip time delay is measured. Figure 4 and Figure 5 show samples and the distribution of the time delay, respectively. The output is affected by a non-Gaussian measurement disturbance (ω). The PDF of ω is given by:

(15) $$\gamma_{\omega }(x)=\{\begin{array}{c} {\left[0.01^{\lambda +\mu +1}\beta (\lambda +1,\mu +1)\right]}^{-1}x^{\lambda }{\left(0.01-x\right)}^{\mu },x\in (0,0.01)\\ 0,\mathrm{otherwise} \end{array}$$

where $\beta (\lambda +1,\mu +1)=\int_{0}^{1}x^{\lambda }{\left(1-x\right)}^{\mu }dx,\lambda =4,\mu =3$.

**Figure 6:**
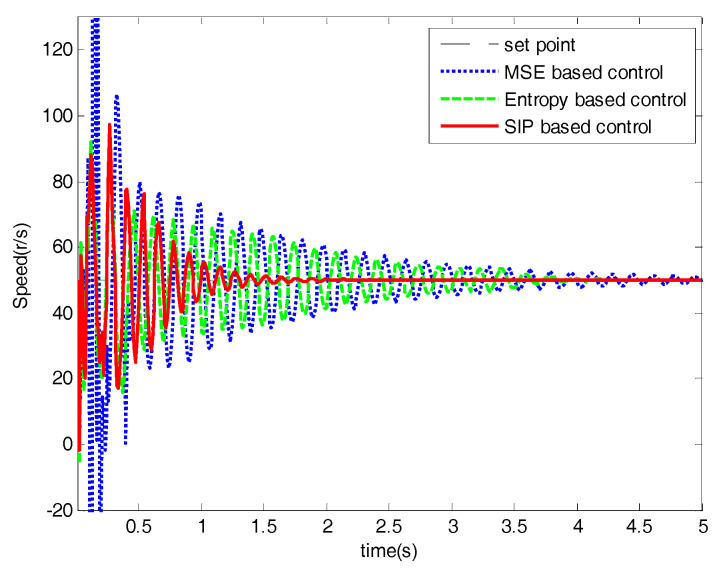
System responses.

**Figure 7:**
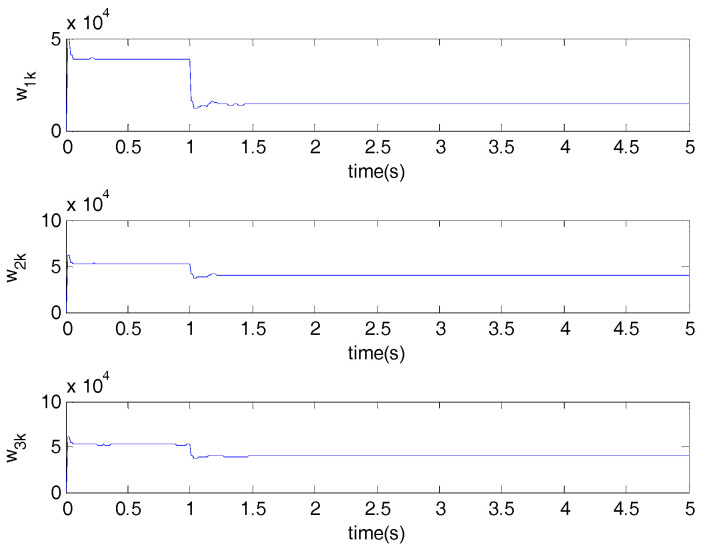
Weights of a single neuron.

**Figure 8:**
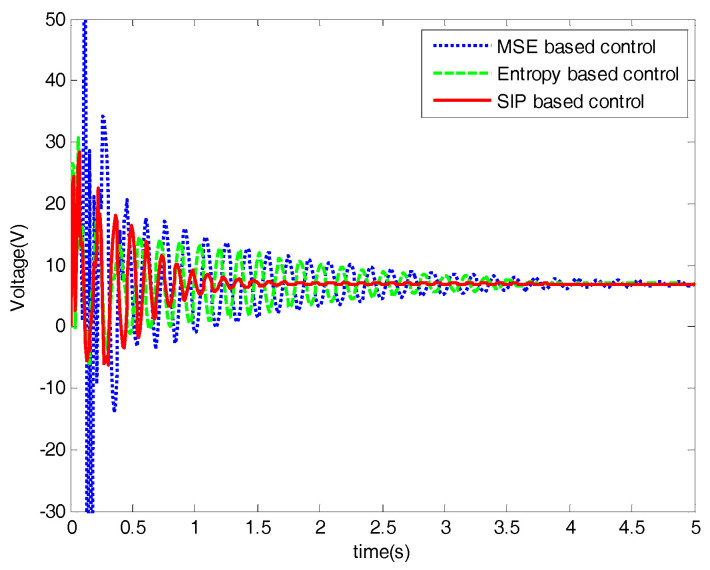
Control inputs.

**Figure 9:**
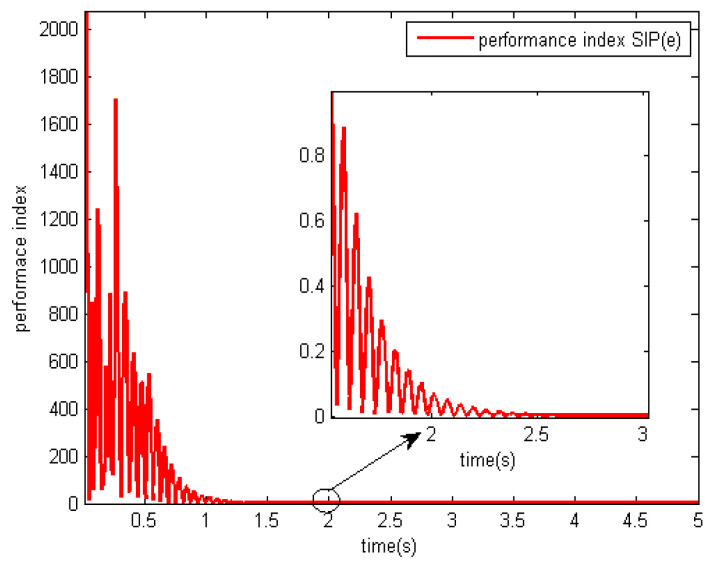
Performance indexes of error with SIP criterion.

**Figure 10:**
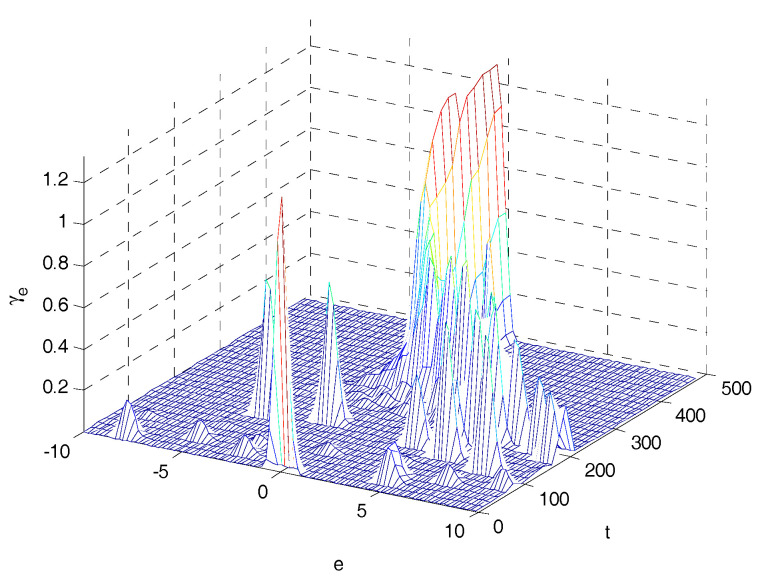
3D mesh PDF using the entropy based controller.

**Figure 11:**
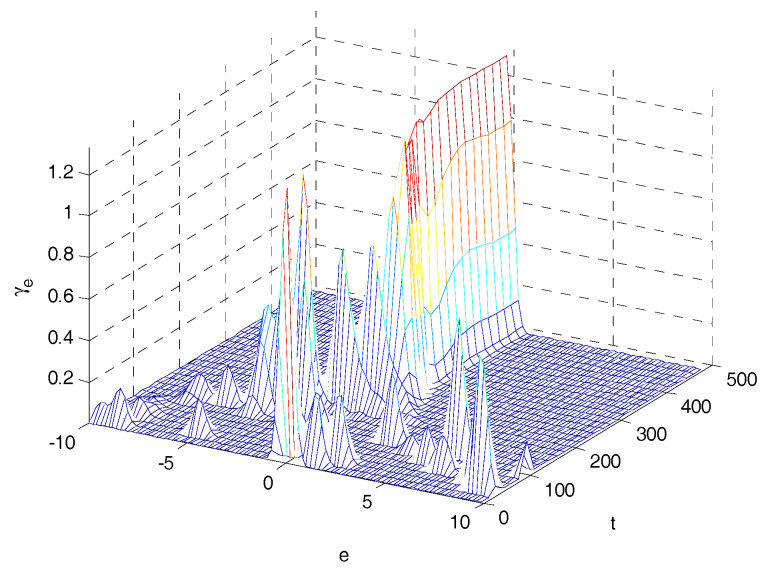
4D mesh PDF using the SIP based controller.

**Figure 12:**
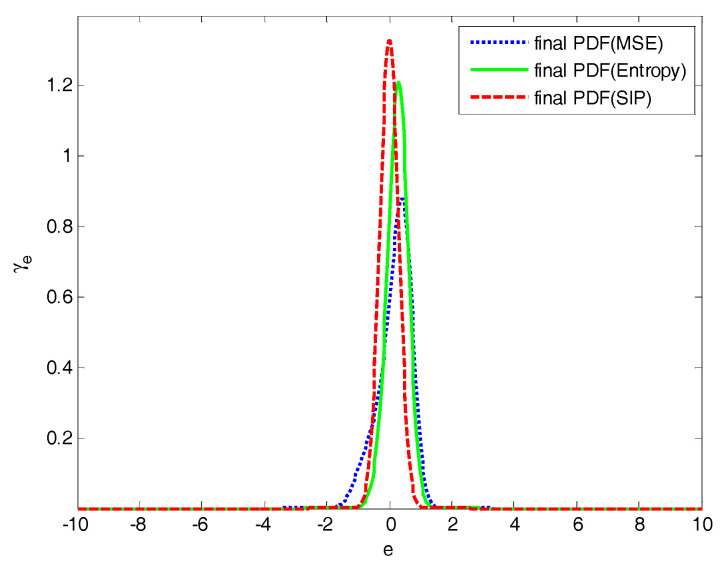
Final PDFs of the tracking error.

Three statistical information indexes, mean value, standard deviation and the SIP value of three control methods are listed in Table 1, which illustrates that the proposed SIP-based control algorithm is better than the entropy-based method. The control performances of the networked DC motor control systems are shown in Figure 6, Figure 7, Figure 8, Figure 9, Figure 10, Figure 11 and Figure 12. 

The performances of the proposed optimal control algorithm based on SIP, MSE and the minimum entropy control (MEE) in controlling a networked DC motor control system are compared. The conventional MSE method could only control well based on the assumption that the system variables follow a Gaussian distribution, but the disturbances and delays are of non-Gaussian types in Figure 3. Therefore, the MEE strategy, instead of MSE, is utilized to deal with non-Gaussian randomness. However, the MEE strategy has some shortcomings as mentioned above, so SIP was proposed to overcome the entropy drawbacks. Figure 6 shows the response of the DC motor speed, the solid line is the response of the DC motor speed using the proposed method. It’s obvious that the SIP-based control method could control well and stabilize the speed around the set point, which has rapid convergence, and its speed fluctuation is small. According to the SIP value from Table 1, the randomness is reduced and approaches zero with the proposed method. Although the fluctuation of speed with the entropy method and SIP value is also small, it converges to the target value at a very slow speed. However, the MSE control strategy could not control well, which has high standard deviation. It is clear that the DC motor speed control system with SIP criterion has superior performance than that based on entropy and MSE controller. Figure 7 shows the weights of the single neuron. Obviously, the required control force under the proposed control law is smaller than that with entropy and MSE seen in Figure 8. Figure 9 demonstrates the SIP error value is decreasing overall with the progress of time, which represents that the control performance is getting better. In order to demonstrate the SIP value is close to zero during the stationary phase, the SIP performance indexes after two moments are showed in the amplification region. 

The 4-D mesh plots of the PDFs of tracking error are shown in Figure 10 and Figure 11. Comparing Figure 10 with Figure 11, the shape of the PDFs of tracking error in Figure 10 becomes narrower and sharper along with sampling time; it indicates that the proposed control system has a small uncertainty in its closed loop operation. The final PDF of three control laws are presented in Figure 12, in which PDFs of proposed method is sharper than that under MSE law and entropy-based control law, which indicates that the proposed control strategy can obtain better performance. 

## 6. Conclusions

In this work, a new single neuron control methodology for networked systems with non-Gaussian delay and disturbances is presented. Instead of the entropy criterion, a SIP-based performance function is adopted to design the controller, and the tracking control problem is converted into an optimization one. In order to calculate the tracking error SIP, the oversampling method is used here. For simplicity, the optimization problem is solved by using the well-known and effective gradient descent method. The proposed control strategy is applied in a networked DC motor control system. It is confirmed from the simulation results that the proposed SIP-based predictive strategy can achieve a better tracking performance than MSE and entropy. To sum up, the proposed optimal control algorithm has the following advantages:

(1) This strategy is data-driven, which can avoid modeling error and complexity in building the model.
(2) A general measure, SIP, is used to formulate the performance index to design the control algorithm, which could overcome shortcomings of entropy.
(3) Oversampling method is used to calculate the SIP of tracking error, which is more practical for online controller design problem.

Extending SIP to Multiple-Input-Multiple-Output (MIMO) NCS is a relevant research problem. In the MIMO system, there is an interaction between the variables. Some preprocessing of output variables should be done to eliminate multi-colinearity. Extending our approach to MIMO systems will be included in our future research topics. 

## Figures and Tables

**Table 1:** Control performance indexes.

Disturbances	Method	The Mean Value	The Standard Deviation	The SIP Value
Non-Gaussian	MSE	49.9153	22.6583	3.0000
	Entropy	49.6720	10.9471	0.4997
	SIP	49.8978	8.1570	0.1054
